# Emerging therapeutic agents for lung cancer

**DOI:** 10.1186/s13045-016-0365-z

**Published:** 2016-12-09

**Authors:** Bhagirathbhai Dholaria, William Hammond, Amanda Shreders, Yanyan Lou

**Affiliations:** Department of Hematology-Oncology, Mayo Clinic, 4500 San Pablo Road, Jacksonville, FL 32224 USA

**Keywords:** Lung cancer, Targeted agents, Immunotherapy, Phase I/II clinical trial

## Abstract

Lung cancer continues to be the most common cause of cancer-related mortality worldwide. Recent advances in molecular diagnostics and immunotherapeutics have propelled the rapid development of novel treatment agents across all cancer subtypes, including lung cancer. Additionally, more pharmaceutical therapies for lung cancer have been approved by the US Food and Drug Administration in the last 5 years than in previous two decades. These drugs have ushered in a new era of lung cancer managements that have promising efficacy and safety and also provide treatment opportunities to patients who otherwise would have no conventional chemotherapy available. In this review, we summarize recent advances in lung cancer therapeutics with a specific focus on first in-human or early-phase I/II clinical trials. These drugs either offer better alternatives to drugs in their class or are a completely new class of drugs with novel mechanisms of action. We have divided our discussion into targeted agents, immunotherapies, and antibody drug conjugates for small cell lung cancer (SCLC) and non-small-cell lung cancer (NSCLC). We briefly review the emerging agents and ongoing clinical studies. We have attempted to provide the most current review on emerging therapeutic agents on horizon for lung cancer.

## Background

Lung cancer is the second most commonly diagnosed cancer and is the leading cause of cancer-related death in both men and women. Implementation of tobacco control, low-dose spiral computed tomography screening programs, and advances in multidisciplinary treatments have resulted in the slow decline of both incidence and mortality. However, 52–58% of lung cancer patients present with advanced-stage disease, and a vast majority of these patients do not survive despite treatment. Similarly, the prognosis remains poor even in locally advanced disease because of the high relapse rate and early formation of micrometastases [1].

**Fig. 1:**
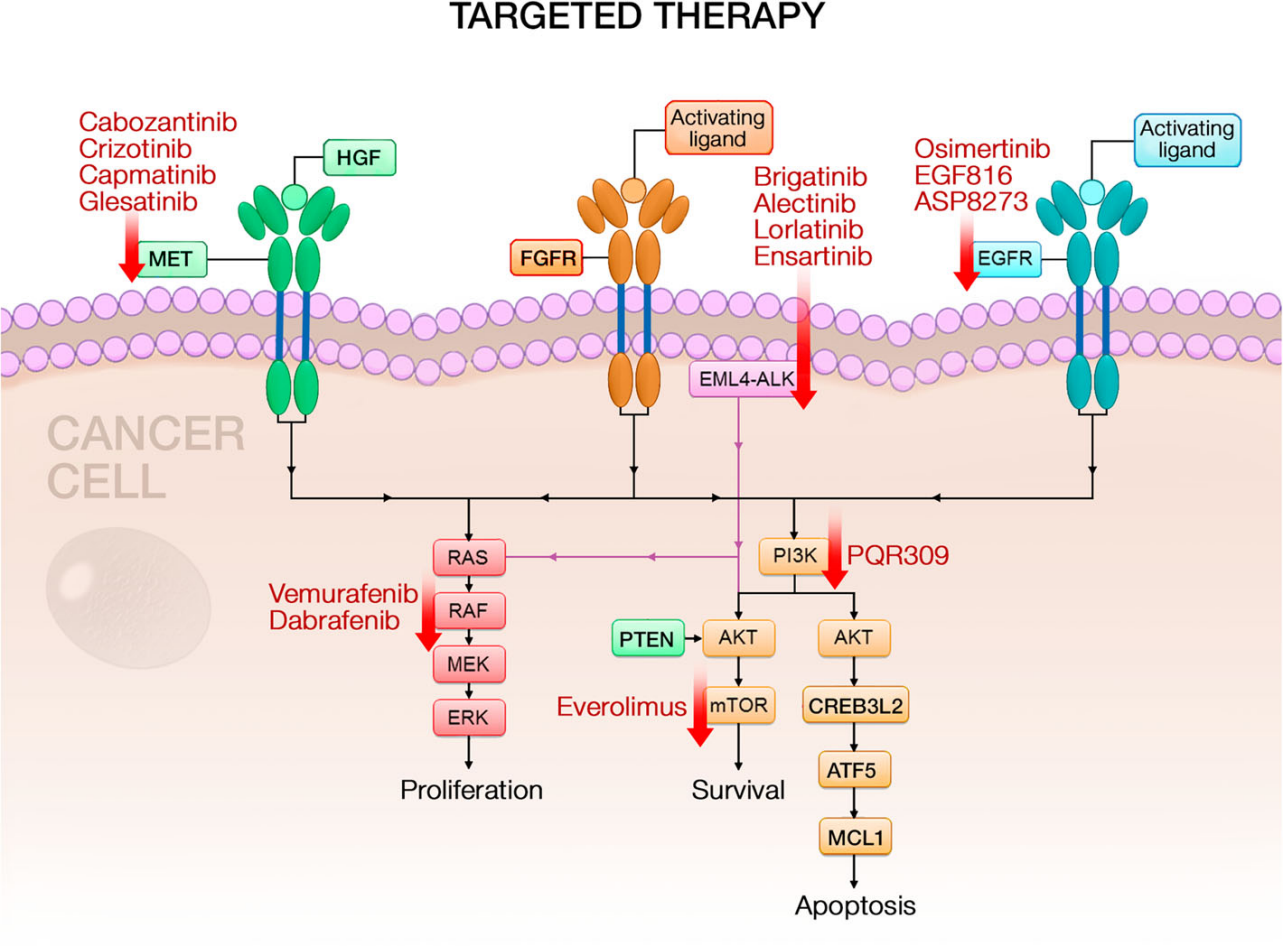
Molecular targets and inhibiting agents being studied in phase I/II trials as potential therapy for patients with lung cancer. Abbreviations: *AKT* protein kinase B, *ALK* anaplastic lymphoma kinase, *CREB3L2* cyclic AMP-responsive element-binding protein 3-like protein 2, *EGFR* epidermal growth factor receptor, *EML4* echinoderm microtubule-associated protein-like 4, *ERK* extracellular signal-regulated kinase, *FGFR* fibroblast growth factor receptor, *HGF* hepatocyte growth factor, *MCL1* myeloid leukemia cell differentiation protein, *MEK* mitogen-activated protein kinase, *MET* mesenchymal-to-epithelial transition, *mTOR* mammalian target of rapamycin, *PTEN* phosphatase and tensin homologue, *RAF* rapidly accelerated fibrosarcoma kinase, *RET* rearranged during transfection proto-oncogene

One of the most important therapeutic advances of lung cancer treatment in the last decade was identification of specific driver mutations and the development of small molecular tyrosine kinase inhibitors (TKIs) [2]. In 2009, erlotinib was the first selective epidermal growth factor receptor (EGFR) inhibitor approved by the US Food and Drug Administration (FDA) [3]. This was quickly followed by crizotinib, which was initially developed as a MET (mesenchymal-to-epithelial transition/hepatocyte growth factor receptor) inhibitor and was found to be highly active against small subset of non-small-cell lung cancer (NSCLC) cases harboring anaplastic lymphoma kinase (ALK) rearrangement [4]. These drugs promise around a 70% response rate; however, resistance development is almost universal, and second/third generation TKIs are being developed to overcome these issues. Novel targeted agents directed against EGFR, ALK, ROS1, MET, RET, BRAF, and many more are under investigation. Figure 1 provides summary of targets with specific focus on the drugs that currently in early-phase clinical trials in lung cancer. In addition to these drugs, next-generation sequencing and cell-free DNA (cfDNA) technologies have provided rapid and convenient tools for gene abnormality testing and the development of targeted therapies [5]. Additionally, personalized medicine has become part of daily practice, and tailoring treatment for individual patients is becoming a reality.

**Fig. 2:**
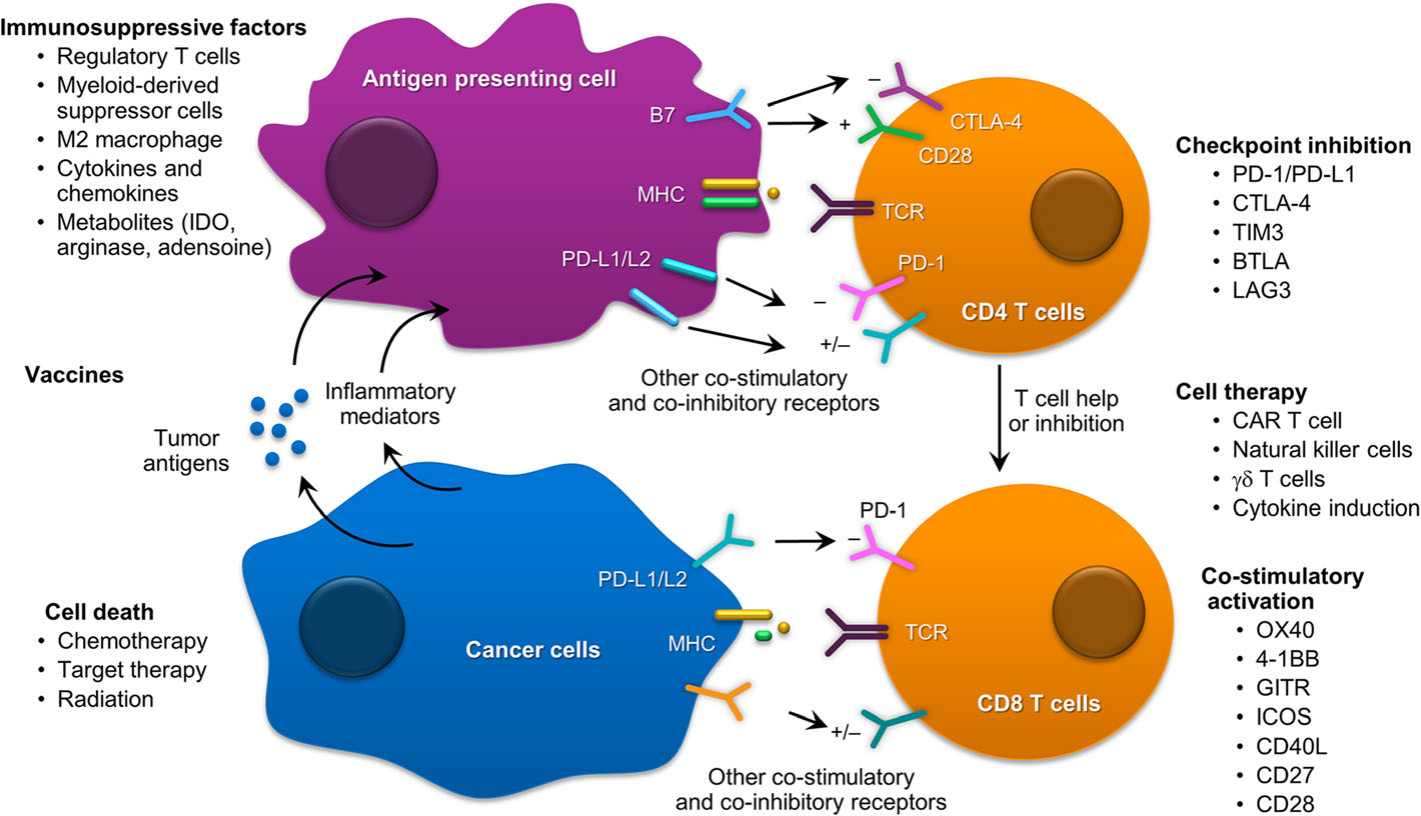
Multifaceted immunotherapy approaches to target cancer cell

Immunotherapy in the form of checkpoint inhibitors represents a landmark success in NSCLC treatment, and patients have experienced durable responses with good tolerability. Pembrolizumab and nivolumab exert antitumor activity by blocking programmed death receptor-1 (PD-1) on T lymphocytes. These drugs are currently approved as second-line therapies for advanced NSCLC based on pilot studies that show improved and durable responses compared to docetaxel [6–8]. Most recently, the FDA approved pembrolizumab for the treatment of patients with metastatic NSCLC whose tumors express strong PD-L1 in the first-line setting based on significant improvement in progression-free survival (PFS) and overall survival (OS) [9]. Trials are underway to test using these agents as first-line therapies for patients with NSCLC either alone or in combination with chemotherapy, TKIs, radiation, and other immunotherapies [9–12]. For example, combinations of CTLA-4 and PD-1 inhibitors have been investigated in patients with NSCLC and small cell lung cancer (SCLC). Preliminary results from a phase I study demonstrated that ipilimumab and nivolumab can be effectively and safely combined as first-line treatment of advanced NSCLC [10]. This combination is currently being tested in ongoing phase III study. Similarly, increased antitumor activity was also seen in SCLC with this combination [11]. Multiple studies are underway to investigate the clinical activities of combined chemotherapy and checkpoint inhibitors. Studies to investigate the roles of checkpoint inhibitors in adjuvant and neoadjuvant settings in early-stage lung cancers are ongoing as well. These exciting developments have fuelled rapid progress in the field, and multiple molecules targeting different aspects of host-tumor immune interactions are currently being investigated. Figure 2 provides the summary of ongoing strategies and efforts in immunotherapy of lung cancer.

In this review, we have discussed recently published data on the first-in-human clinical trials and some of the most promising drugs in pipeline. Literature was searched for phase 1/2, first in human clinical trials in lung cancer by using PubMed, Google scholar, and the American Society of Clinical Oncology (ASCO) meeting abstracts. Each study was individually reviewed and data points have been summarized.

## Targeted agents

### EGFR inhibitors

EGFR is a member of the ErbB tyrosine kinase receptor (TKR) family and is referred to as ErbB1 or HER1. Gefitinib was first tested for EGFR-expressing NSCLC. It targets the ATP cleft within EGFR, which is overexpressed in 40–80% of NSCLC cases. Later, Lynch et al. demonstrated that only the tumors with somatic mutations in tyrosine kinase domain of the *EGFR* gene responded to gefitinib [13]. Testing for driver mutations in newly diagnosed, advanced NSCLC cases has become the standard of care. In patients who carry the targetable driver mutation, a first-line treatment with targeted agents is recommended over conventional chemotherapy. These drugs are well tolerated and give predictable objective response. A phase 2 trial in neo-adjuvant settings has shown an improved response rate compared to chemotherapy in *EGFR*+ NSCLC [14]. Driver mutations in *EGFR* (exon 19 deletion or exon 21 L858R substitution) are found in 15–20% of all lung adenocarcinomas (ACs) that account for the largest group of lung cancer patients. Erlotinib, gefitinib, and afatinib are approved as first-line treatments for targetable *EGFR* alterations. The median progression-free survival (PFS) from these agents is 9.2–13.1 months [15–17]. Dacomitinib is a small molecule, irreversible inhibitor active against all HER family of tyrosine kinases. In randomized trials, it has comparable efficacy to erlotinib. The subgroup with EGFR exon 19 deletion has better PFS with dacomitinib compare to erlotinib (HR 0.585, *P* = 0.058) [18, 19]. A recent phase 3 trial (NCT01040780) comparing icotinib with gefitinib as the first-line EGFR TKI treatment showed similar results in Chinese patients [20].

#### T790M mutant-selective EGFR TKIs

After the initial response to EGFR TKI, resistance development through various mechanisms is inevitable. The *EGFR* T790M mutation causes acquired resistance to the first- and second-generation TKIs. T790M mutation-selective third-generation EGFR TKIs (osimertinib, rociletinib) have been developed with encouraging overall response rates up to 60% [21, 22]. Osimertinib was approved in 2015 by the FDA for confirmed *EGFR* T790M mutation-positive NSCLC. A first-line trial (NCT02296125) is underway to compare osimertinib vs erlotinib or gefitinib. This should give information on ideal sequencing on these agents. ASP8273 and olmutinib (BI1482694) are other third-generation T790M-selective EGFR TKIs. Early-phase studies (NCT02113813, NCT01588145) in T790M-positive NSCLC showed an overall response rate (ORR) of 31% and disease control rate (DCR) of 57% for ASP8273, as well as a 61% ORR and 90% DCR for BI1482694. The median PFS was 6.8 and 8.3 months, respectively [23, 24]. EGF816 is another agent that showed an ORR of 44% and DCR of 91% in a phase 1 study (NCT02108964) [25]. Typical EGFR TKI-related adverse events were observed with all three drugs.

#### CNS-penetrant EGFR TKIs

Central nervous system (CNS) metastasis is a common site of disease progression in *EGFR*+ NSCLC, leading to failure of the first-line TKIs. AZD3759 is a potent CNS-penetrant EGFR TKI currently being evaluated along with osimertinib in a phase 1 (BLOOM) study (NCT02228369) in patients with progressive CNS metastasis who have already had first-line treatment with EGFR TKI. AZD3759 showed activity in 20 patients with measurable brain metastasis evaluable for RECIST assessment; 8 had tumor shrinkage in the brain, 3 had confirmed partial response (PR), and 3 had unconfirmed PR [26]. In 12 patients taking osimertinib, 7 had radiological PR, 2 had stable disease, and 3 were not evaluable after 12 weeks [27].

Epitinib is another small-molecule TKI being developed for its favorable CNS penetration. In a dose-expansion phase, 12 patients with *EGFR*+ NSCLC and CNS metastasis received epitinib. Among those 12 evaluable patients, 5 reached PR (all treatment naïve) and showed dramatic shrinkage of brain lesions. The five prior-TKI-treated patients had stable disease (SD) in the brain [28] (NCT02590952).

### ALK inhibitors

*ALK* fusion oncogene-associated NSCLC is a distinct subset of lung cancer amenable to targeted therapy. *ALK* rearrangement is found in 3–13% of NSCLC cases, with a higher prevalence among younger patients, never or light smokers, and adenocarcinoma with signet ring or acinar histology [29]. Crizotinib is a multi-targeted TKI that is active against *ALK*, *ROS1*, and MET [4]. It has been approved as a first-line treatment for *ALK*+ or *ROS1*+ NSCLC. Ceritinib and alectinib are second-generation ALK TKIs approved for crizotinib-resistant or intolerant cases [30–32]. Resistance development and CNS progression are major issues, and the following ALK TKIs are currently under investigation.

Brigatinib was given to 222 patients with crizotinib-refractory, *ALK*+ NSCLC under a phase II study (ALTA, NCT02094573). A PFS of 8.8 and 11.1 months was noted among those receiving lower and higher doses of brigatinib, respectively. An encouraging intracranial ORR of 67% was seen in nine patients with measurable CNS metastasis. Early onset pulmonary toxicity was seen in 6% of the patients [33]. A phase III trial (NCT02737501) against crizotinib in a front-line setting for *ALK*+ NSCLC has been initiated.

Lorlatinib (PF-06463922) was tested in phase I/II study (NCT01970865) of *ALK*+/*ROS*+ NSCLC. A majority of the participants had a prior treatment with ≥2 ALK TKIs. Among 41 evaluable patients, an ORR 46% and median PFS of 11.4 months were seen. The intracranial ORR was 44%, including a few complete responses (CRs) [34]. Interestingly, the drug was active against diseases caused by the *ALK* G1202R mutation which confers resistance to ceritinib, alectinib, and brigatinib [35].

Ensartinib (X-396) is a novel ALK inhibitor with additional activity against ROS1, MET, SLK, Axl, LTK, ABL, and EPHA2. Partial responses were seen in 60% crizotinib-naïve (30 patients) and 88% crizotinib-resistant patients (12 patients) (NCT01625234). CNS responses were also observed in both groups [36]. A phase 3 study (NCT02767804) comparing ensartinib and crizotinib in a front-line setting is currently recruiting patients.

### MET inhibitors

MET, a receptor tyrosine kinase after binding with hepatocyte growth factor (HGF), activates the phosphatidylinositol-3-kinase (PI3K) and mitogen-activated protein kinases (MAPK) pathways. *MET* gene amplification and exon-14-skipping mutations are characteristic abnormalities causing increased MET signaling activation. Isolated *MET* exon 14 mutation is found in 3% of NSCLC; however, it is an acquired EGFR TKI resistance pathway in 15–20% of *EGFR* mutation-positive NSCLC cases [37, 38]. Crizotinib has shown some activity in selected *MET*-amplified and exon 14-skipping mutant NSCLC [39, 40] (NCT00585195). Cabozantinib, a multi-targeted MET inhibitor, was given to five patients with exon 14 mutations and had a stable disease for 5 months [40]. Capmatinib (INC280) is a selective MET inhibitor. In a phase I study (NCT01324479), relapsed NSCLC patients with high cMET expression were given capmatinib. In subgroup of patients with *MET*-amplified disease, the ORR was 63%, and the median PFS was 7.4 months [41]. Glesatinib (MGCD265) is another *MET* blocker currently being studied in NSCLC (NCT00697632).

#### Combined MET + EGFR inhibitors

Acquired EGFR TKI resistance is mediated by *MET* upregulation in a subset of NSCLC patients. One strategy to overcome this resistance is to combine a MET inhibitor with an EGFR TKI. A combination of capmatinib and gefitinib was tested in a phase 2 study (NCT01610336) in *EGFR*+ NSCLC patients after their disease progressed while using gefitinib. *EGFR* T790M NSCLCs were excluded and high cMET expression was required. An ORR of 18%, an SD of 62%, and a DCR of 80% were observed in 65 evaluable patients. More responses were seen in tumors with *MET* amplifications [42]. Tepotinib (MSC2156119J) with gefitinib was well tolerated in a phase 1 study (NCT01982955). Eighteen patients were treated, and five had a partial response [43]. A similar trial (NCT01900652) with emibetuzumab and an IgG4 anti-MET monoclonal antibody (mAb) with erlotinib in MET-expressing NSCLC with acquired erlotinib resistance showed some benefit in high cMET-expressing tumors [44].

### RET inhibitors

The *RET* proto-oncogene encodes a receptor tyrosine kinase for members of the glial cell line-derived neurotrophic factor (GDNF) family. *RET* rearrangements are found in 1–2% lung adenocarcinoma and are mutually exclusive with mutations involving *EGFR*, *ALK*, or *KRAS*. Vandetanib, sorafenib, sunitinib, lenvatinib, ponatinib, and cabozantinib are multi-targeted TKIs with RET-blocking activity. They are currently approved for other malignancies. In a phase 2 study with cabozantinib, 38% PR was seen among 16 evaluable patients, and there was a median PFS of 7 months [45].

Vandetanib in advanced *RET*-rearranged NSCLC showed an ORR 53%, a DCR 88%, and a median PFS of 4.7 months in 17 eligible patients. The CCDC6-*RET* subtype had an ORR of 83% and a median PFS of 8.3 months [46] (UMIN000010095). In a phase 1 study (NCT01582191), vandetanib was combined with everolimus (mTOR (mammalian target of rapamycin) inhibitor) to prevent resistance development based on in vitro studies. Among 13 stage IV NSCLC patients, PR was achieved in five patients with *RET* fusion. Good CNS activity was seen in three patients with intracranial metastasis [47].

### BRAF/MEK inhibitors

BRAF is a downstream signaling mediator of KRAS, which activates the MAP kinase pathway. *BRAF* mutations are found in 1–2% of NSCLC cases and are usually smoking related [48]. It is also described as one of the resistance mechanisms associated with EGFR TKIs. Vemurafenib and dabrafenib are currently approved for *BRAF* V600E-positive malignant melanomas, but single-agent activity in *BRAF* V600E- positive NSCLC is limited. Dabrafenib had an ORR of 33% in platinum refractory cases with a median duration of response of 9.6 months in single study [49] (NCT01336634).

#### Combination BRAF + MEK inhibitors

Sequential inhibition of BRAF and downstream MEK is an active area of lung cancer research after encouraging results were seen in melanoma patients. Dabrafenib with trametinib (MEK inhibitor) was associated with ORR of 63% in 57 evaluable patients and the DCR was 79% [50].

#### Combination MEK inhibitor and immunotherapy

MEK inhibition can result intratumoral T cell accumulation and MHC-1 upregulation and synergizes with anti-PD-L1 agent leading to tumor regression [51]. Cobimetinib is a selective MEK1 and MEK2 inhibitor. In a phase 1b study (NCT01988896), cobimetinib with atezolizumab (anti-PD-L1) was given to advanced solid cancer patients. In colorectal cancer cohort (MSI-low), ORR was 17% and responses were durable. Increased PD-L1 and CD8 T lymphocyte infiltration was demonstrated in serial biopsies [52]. Results on NSCLC cohort are pending.

### PI3K inhibitors

Phosphatidyl 3-kinase (PI3K) pathway is a central mediator of cell survival signals. *PI3CA* mutations are found in 4.4% of lung adenocarcinoma and 16% of squamous cell cancer*. PI3CA* amplifications are found in up to 40% of squamous cell lung cancer [53, 54]. *PI3CA* mutations also promote resistance to EGFR TKIs. PQR309 is a pan-PI3K, mTOR inhibitor. Its safety and maximum tolerated dose have been recently established in a phase 1 study (NCT02483858) with advance solid cancers. No response data are available for NSCLC cohort [55].

### HER3 inhibitors

Patritumab is a fully human anti-HER3 mAb. HER3 is a member of the ErbB tyrosine kinase receptor family and is activated by heregulin. Preclinical studies with EGFR+ NSCLC cell lines have shown that increased heregulin level confers resistance to EGFR TKI. An initial phase 2 study (NCT01211483) failed to show the PFS benefit of adding patritumab to erlotinib compared to a placebo. However, the subgroup with increased soluble heregulin showed increased PFS (HR 0.41 [95% CI 0.18–0.90], *P* = 0.02) with patritumab [56]. A phase 3 placebo-controlled study (HER3-Lung, NCT02134015) in *EGFR*+ NSCLC is ongoing.

### Aurora A kinase inhibitors

Aurora A is a member of a family of mitotic serine/threonine kinases which assist with cell proliferation. Alisertib (MLN-8237) is an aurora kinase A inhibitor. Based on in vitro synergism with EGFR TKIs, it was tested in 18 patients with EGFR+ NSCLC. The combination was well tolerated; one patient had a PR, while five other patients achieved SD [57]. Phase 2 recruitment is ongoing (NCT01471964).

### FGFR inhibitors

The fibroblast growth factor receptor (FGFR) binds to members of the fibroblast growth factor family of proteins and promotes cell proliferation. BGJ398 is a potent, selective pan-FGFR (fibroblast growth factor receptor). *FGFR1* amplification is found in around 21% of squamous NSCLC cases [58]. In a single phase 2 trial, 26 evaluable patients showed a PR of 15% and an SD if 35% in dose >100 mg [59]. Dovitinib is another FGFR inhibitor tested in squamous NSCLC. Among 26 patients, the ORR was 11.5%, the DCR was 50%, and the median PFS was 2.9 months [60].

### PARP inhibitors

PARP (poly ADP ribose polymerase) plays an important role in DNA repair. PARP inhibitors have synergistic activity with platinum based chemotherapy (i.e., cisplatin) [61]. In a phase 2 study, veliparib in combination with carboplatin and paclitaxel was tested in first-line setting for advanced NSCLC. The combination was well tolerated, and there was a trend toward favorable PFS (HR 0.72 [95% CI 0.45–1.15, *P* = 0.17]) and OS (HR 0.80 [95% CI 0.54–1.18, *P* = 0.27]) in combination arm [62]. A phase 2 combining veliparib with chemoradiation in stage 3 NSCLC is recruiting (NCT02412371).

## Immunotherapies

Immunotherapy has long been considered the holy grail of Oncology. Different attempts have been made to harness body’s immune system to eradicate malignancy. Lung cancer has the second highest mutation burden after melanoma, which increases its susceptibility to immunotherapy [63]. One of the key advantages of immunotherapy is the durability of responses. Where resistance development is universal with chemotherapy and targeted agents, memory function of the immune system can lead to lasting remission. Check point inhibitors target the PD1/PD-L1 axis to remove the inhibitory signals on T lymphocytes to eradicate malignancy. Pembrolizumab and nivolumab are currently approved as second-line therapies for both adenocarcinoma and squamous cell carcinoma of lung. Atezolizumab is the first anti-PD-L1 agent approved by the FDA for NSCLC in second-line setting after encouraging results from phase 3 OAK trial showing superior OS.(HR 0.73; *P* = 0.0003) [64]. A recent phase 3 study tested pembrolizumab vs platinum doublet chemotherapy in first-line NSCLC with at least 50% PD-L1expression in tumor cells. Pembrolizumab was associated with better PFS (10.3 vs 6 months) and response rate (44.8 vs 27.8%) compared to chemotherapy. Estimated survival at 6 months was also better in patients treated with pembrolizumab (HR = 0.60, *P* = 0.005) [9]. This had led to approval of pembrolizumab as first-line therapy in NSCLC with >50% PD-L1 expression in tumor cells. A combination of ipilimumab and nivolumab as first-line treatment in advanced NSCLC is feasible, safe, and has shown good ORR [10]. Durvalumab (anti-PD-L1) and tremelimumab (anti-CTLA4) have been tested in phase 1b study and found to be safe with early evidence of clinical activity in relapsed NSCLC [65]. As these agents move quickly to treat early-stage lung cancers through clinical studies, other agents that target different aspects of the tumor-immune system milieu are being developed. Figure 2 demonstrates different strategies being utilized to enhance tumor recognition and elimination by the immune system [66].

### OX40

OX40 (CD134) is a secondary costimulatory immune checkpoint receptor expressed on activated T cells that lead to expansion of effector and memory T cells after binding with OX40L (CD252) on antigen presenting cells. It is a part of tumor necrosis factor receptor (TNFR) superfamily and works in conjunction with B7 family members, such as PD1 and CTLA-4. GSK3174998 is a humanized IgG1 agonistic anti-OX40 monoclonal antibody (mAb) that promotes naïve CD4+ T- cells and suppresses their differentiation into immunosuppressive regulatory T cells (T_reg_) [67, 68]. ENGAGE-1 (NCT02528357) is a first-in-human study of GSK3174998 alone and in combination of pembrolizumab. Murine studies have shown synergistic activity between PD-1 blockade and OX40 agonist mAb [69]. No dose-limiting toxicity was found in the initial dose escalation cohort alone and in combination with a 200-mg fixed dose of pembrolizumab every 3 weeks. This study is still recruiting patients.

MEDI6383 (NCT02221960) is another OX40 agonist mAb currently being studied with durvalumab, an anti-PD-1 antibody. Lirilumab/BMS-986015 (NCT01714739) is anti-KIR mAb, which is a natural killer (NK)-cell checkpoint inhibitor that is being evaluated with nivolumab.

### Lymphocyte activation gene 3

Lymphocyte activation gene 3 (LAG-3, CD223) is another checkpoint inhibitor on activated T lymphocytes that inhibits immune function after binding with major histocompatibility complex (MHC) II [70]. Urelumab is an anti-LAG3 mAb currently being studied for NSCLC in combination with anti-PD1 agents (NCT01968109, NCT02460224).

### T cell immunoglobulin and mucin-domain containing-3

T cell immunoglobulin and mucin-domain containing-3 (TIM-3) is Th1 (T helper-1)-specific regulator of macrophage responses. Recently, murine studies showed TIM-3 upregulation in the tumor microenvironment in an anti-PD1 resistant NSCLC model [71]. MBG453 and TSR-022 are anti-TIM3 mAbs currently being studied in two phase 1 trials (NCT02817633, NCT02608268).

### B7-H3

B7-H3 is widely expressed among tumors and is associated with immune escape and metastasis. MGD009 is a humanized B7-H3 and CD3 dual-affinity re-targeting (DART) protein designed to engage T cells to B7-H3 expressing cells. Antitumor activity in multiple in vivo models has been demonstrated. A phase 1 trial (NCT02628535) in advanced B7-H3 expressing tumors, including NSCLC, is recruiting patients [72]. Prior anti-PD1 therapy is allowed.

### MUC1

MUC1 is widely expressed in many malignancies, including NSCLC. TG4010 is a modified vaccinia Ankara expressing MUC1 and interleukin 2 (IL2). In a recent phase 2b study, TG4010 or placebo were given with chemotherapy as a first-line therapy for metastatic NSCLC (mNSCLC) without EFGR mutations and MUC1 expression in minimum 50% of tumor cells. Significant improvement in PFS (hazard ratio 0.74, 95% CI 0.55–0.98, *P* = 0.019) was noted. The phase 3 component is ongoing [73].

### GM.CD40L

GM.CD40L is an allogeneic tumor cell vaccine developed from a human bystander cell line. Cells are transduced with granulocyte-macrophage colony-stimulating factor (*GM-CSF*) and CD40-ligand (*CD40L*) genes. In vivo, it stimulates dendritic cell-mediated immune response. CCL21 is a chemokine that helps with T cell responses. In a phase 1/2 study (NCT01433172), GM.CD40L + CCL21 did not improve PFS, and the median OS was comparable to single-agent chemotherapy in advanced adenoNSCLC. A combination study with anti-PD1 is underway [74].

## Antibody drug conjugates

Antibody drug conjugates (ADCs) are an important class of biologics currently being investigated for range of malignancies. The chemotherapy molecule is attached to a target-specific antibody for tumor-directed cytotoxicity but sparing of normal tissue. Brentuximab vedotin (CD30 directed) and transtuzumab emtansine (HER2 directed) are ADCs currently available for relapsed Hodgkin lymphoma and metastatic HER2-positive breast cancer, respectively. The following ADCs are currently being studied in phase 1 trial for lung cancer.

### Sacituzumab govitecan (IMMU-132)

Trop-2 is widely expressed among solid tumors. Sacituzumab govitecan is made from a humanized anti-Trop-2 monoclonal antibody (hRS7) that is conjugated with the active metabolite of irinotecan, SN-38. In a phase 1/2 study (NCT01631552), the single-agent sacituzumab govitecan was given to patients with advanced epithelial malignancies. In the NSCLC cohort, an objective response of 31% and median PFS of 3.9 months was observed. The SCLC cohort showed a similar response rate, a median PFS of 4.6 months, and a median OS of 8.3 months [75, 76]. Diarrhea and neutropenia were common adverse events. These are encouraging results in platinum refractory patients. It has received the FDA breakthrough therapy designation for triple-negative breast cancer.

### Rovalpituzumab tesirine (SC16LD6.5)

Rovalpituzumab tesirine also known as Rova-T is an antibody (SC16) targeting delta-like protein 3 (DLL3) and is linked to pyrrolobenzodiazepine (PBD). DLL3, an inhibitory Notch ligand, is expressed in >80% of SCLCs. The first biomarker directed trial for advanced SCLC showed an objective response rate of 18% and a clinical benefit rate of 68% as a second or third line of therapy in evaluable patients. Interestingly, 27 patients with high DLL3-tumor expression had a response rate of 44 and 45% in the second- and third-line settings, respectively [77]. Thrombocytopenia, a skin rash, and serosal effusions were dose-limiting toxicities. A phase 2 study (TRINITY; NCT02674568) is recruiting patients with DLL3-expressing SCLC.

## Cancer stemness inhibitors

Cancer stem cells are highly resistant to traditional therapies and cause disease relapse after initial response. Napabucasin (BBI608) is a first-in-class cancer stemness inhibitor that works through STAT3 pathway inhibition. It has shown activity in combination with paclitaxel. In a phase 2 study (NCT01325441), 27 heavily pretreated NSCLC patients were given a combination of napabucasin and weekly paclitaxel. Among 19 evaluable patients, there was a PR of 16%, a DCR of 79%, and a median PFS of 4 months [78].

Demcizumab (VS-6063) is a humanized IgG_2_ anti-DLL4 (delta-like ligand 4) antibody that inhibits tumor growth by suppressing the Notch pathway. A phase 1b study (NCT01189968) tested demcizumab with carboplatin and pemetrexate as first-line therapies for lung adenocarcinoma. Results showed complete responses in one (3%) of 40 patients and partial responses in 19 (47%) patients [79]. A randomized, placebo-controlled phase 2 trial (DENALI) (NCT02259582) has opened for first-line non-squamous NSCLC. Another study (NCT01859741) of first-line chemotherapy for SCLC is still recruiting patients.

Tarextumab (OMP-59R5) is a human anti-Notch 2/3 receptor mAb. In the phase 1b portion of the PINNACLE study, tarextumab was given with etoposide/platinum chemotherapy in the first-line extensive stage SCLC. The combination was well tolerated. The RECIST response was 77% with a median PFS and OS of 4.4 and 10.4 months, respectively, in 27 treated patients [80]. A randomized study (NCT01859741) is ongoing.

## Future directions

We discussed results of first-in-human phase I/II clinical trials with novel agents in lung cancer in this article. There are many new molecules which are currently being studied for a variety of targets. Histone deacetylase (HDAC) inhibitors and DNA hypomethylating agents target epigenetics for tumor growth suppression. In immunotherapy, new peptide vaccines targeting novel tumor antigens, alternative checkpoint inhibitors, and chimeric antigen receptor T cells (CAR-T) are being developed for the patients who have failed or intolerant to anti-PD1/anti-PD-L1 therapy. Novel small molecular inhibitors directed to inhibit variety of signaling pathways are being developed to overcome resistance to currently available targeted therapies. Table 1 summarizes currently open phase I/II clinical trials for lung cancer patients with pending results. The information in this table was collected from Clinicaltrils.gov (accessed on September, 2016). It can serve as a guide for clinicians who treat relapsed refractory lung cancer.

**Table 1:** Currently open phase I/II clinical trials for lung cancer

Drug class	Drug	Mechanism of action	Clinical trials (phase)	Study design	Disease
Tumor epigenetics	Entinostat	Histone deacetylase (HDAC) inhibitor	NCT02437136 (1/2)	Combination	NSCLC
	HBI-8000		NCT02718066 (1/2)	Combination	NSCLC
	ACY 241		NCT02635061 (1)	Combination	NSCLC
	Epacadostat		NCT02298153 (1)	Combination	NSCLC
	Mocentinostat		NCT02805660 (1/2)	Combination	NSCLC
	CC-486	DNA hypomethylation	NCT02250326 (2)	Combination	NSCLC
	Azacitidine		NCT02009436 (1)	Monotherapy	NSCLC
	RRX-001	DNA methylation, histone deacetylation, and lysine demethylation	NCT02489903 (2)	Monotherapy	NSCLC
Tumor metabolism	Ethaselen	Thioredoxin reductase	NCT02166242 (1)	Monotherapy	NSCLC
	TAS-114	dUTPase	NCT02855125 (2)	Combination	NSCLC
	ADI-PEG 20	Pegylated arginine deiminase	NCT02029690 (1)	Combination	NSCLC
	CCT245737	Checkpoint kinase 1	NCT02797977 (1)	Combination	NSCLC/SCLC
	LY2606368		NCT02860780 (1)	Combination	NSCLC/SCLC
	CB-839	Glutaminase	NCT02771626 (1/2)	Combination	NSCLC
Immunotherapy	Cancer vaccines				
	CV301	Tumor peptide vaccine	NCT02840994 (1/2)	Combination	NSCLC
	TG4010		NCT02823990 (2)	Combination	NSCLC
	Gemogenovatucel-T		NCT02639234 (2)	Combination	NSCLC
	CMB305		NCT02387125 (1)	Monotherapy	NSCLC
	DC-CIK	Dendritic cell vaccine	NCT02688686 (1/2)	Monotherapy	NSCLC
	DCVAC/LUCA		NCT02470468 (1/2)	Monotherapy	NSCLC
	AGS-003-LNG		NCT02662634 (2)	Monotherapy	NSCLC
	JNJ-64041757	Listeria vaccine	NCT02592967 (1)	Monotherapy	NSCLC
	ADXS11-001		NCT02531854 (2)	Combination	NSCLC
	DSP-7888	WT1 vaccine	NCT02498665 (1)	Monotherapy	NSCLC
	S-588410	(HLA)-a*2402-restricted epitope peptides	NCT02410369 (2)	Monotherapy	NSCLC
	AD-MAGEA3 and MG1-MAGEA3	MAGE-A3-expressing maraba virus	NCT02879760 (1/2)	Monotherapy	NSCLC
	L-DOS47	Immunoconjugate	NCT02340208 (1/2)	Monotherapy	NSCLC
			NCT02309892 (1)	Monotherapy	NSCLC
	DRibbles		NCT01909752 (2)	Monotherapy	NSCLC
	Checkpoint inhibitors				
	Enoblituzumab (MGA271)	B7-H3 antibody	NCT02475213 (1)	Combination	NSCLC
			NCT01391143 (1)	Monotherapy	NSCLC
	MGD009		NCT02628535 (1)	Monotherapy	NSCLC
	CM-24	CEACAM1 antibody	NCT02346955 (1)	Combination	NSCLC
	Indoximod	Indoleamine 2,3-dioxygenase (IDO) inhibitor	NCT02460367 (1/2)	Combination	NSCLC
	AMG 820	Colony-stimulating factor 1 receptor (CSF1R)	NCT02713529 (1/2)	Combination	NSCLC
	PF-05082566	4-1BB agonist	NCT02315066 (1)	Combination	NSCLC/SCLC
	PBF-509	Adenosine A2a	NCT02403193 (1/2)	Combination	NSCLC
	CPI-444		NCT02655822 (1)	Combination	NSCLC
	PF-04518600	Anti-OX40 mAb	NCT02315066 (1)	Combination	NSCLC/SCLC
	JNJ-61610588	Anti-VISTA	NCT02671955 (1)	Monotherapy	NSCLC/SCLC
	PDR001	Anti-PD1	NCT02460224 (1/2)	Combination	NSCLC/SCLC
	CA-170	Oral PDL1/PDL2/VISTA inhibitor	NCT02812875 (1)	Monotherapy	NSCLC/SCLC
	Avelumab	PD- L1 inhibitor	NCT02584634 (2)	Combination	NSCLC
	Varlilumab	Anti-CR27 mAb	NCT02335918 (1)	Combination	NSCLC
	JNJ-64457107	Anti- CD40	NCT02829099 (1)	Monotherapy	NSCLC/SCLC
	Modified T cell therapy				
	TIL	Tumor infiltrating lymphocytes	NCT02133196 (2)	Monotherapy	NSCLC
	DC-CTL	Combined dendritic cells- cytotoxic t lymphocyte	NCT02766348 (2)	Monotherapy	NSCLC
			NCT02886897 (1/2)	Combination	NSCLC
	IMMUNICELL®	Autologous γδ- T lymphocytes	NCT02459067 (2/3)	Monotherapy	NSCLC
	MAGE A10^c796^T	Chimeric antigen receptor T lymphocytes	NCT02592577 (1/2)	Monotherapy	NSCLC
	NY-ESO-1^c259^T		NCT02588612 (1/2)	Monotherapy	NSCLC
	ANTI-MUC1 CAR T		NCT02587689 (1/2)	Monotherapy	NSCLC
	PD1 knockout cells	Modified T cell therapy	NCT02793856 (1)	Monotherapy	NSCLC
	Targeted NK cells	Modified NK cell therapy	NCT02118415 (2)	Monotherapy	NSCLC
			NCT02845856 (1/2)	Combination	NSCLC
	WT1-TCRC4-T cells	WT1 targeted t cells	NCT02408016 (1/2)	Monotherapy	NSCLC
	Cytokines				
	rSIFN-co	Recombinant interferon	NCT02387307 (1)	Monotherapy	NSCLC
	ALT-803	IL- 15 agonist	NCT02523469 (1/2)	Combination	NSCLC
	AM0010	Pegylated IL-10	NCT02009449 (1)	Monotherapy	NSCLC
	AAT-007	Prostaglandin E receptor subtype 4	NCT02538432 (2)	Monotherapy	NSCLC
	Poly-ICL	Toll-like receptor agonist	NCT02661100 (1/2)	Combination	NSCLC/SCLC
	VTX-2337		NCT02650635 (1)	Monotherapy	NSCLC
	L19-IL2	Antibody cytokine fusion protein	NCT02735850 (2)	Combination	NSCLC
	CDX-1401	DEC-205/NY-eso-1 fusion protein	NCT02661100 (1/2)	Combination	NSCLC/SCLC
Targeted therapy	EGFR inhibitors				
	ABBV-221	EGFR	NCT02365662 (1)	Monotherapy	NSCLC
	AC0010MA	EGFR T790M	NCT02448251 (1/2)	Monotherapy	NSCLC
	Tesevatinib	EGFR (CNS penetrant)	NCT02616393 (2)	Monotherapy	NSCLC
	JNJ-61186372	EGFR/MET bispecific mAb	NCT02609776 (1)	Monotherapy	NSCLC
	AP32788	EGFR exon 20	NCT02716116 (1/2)	Monotherapy	NSCLC
	MM-151 and MM-121	EGFR mAb	NCT02538627 (1)	Monotherapy	NSCLC
	TargomiRs	EGFR ab bound mir-16	NCT02369198 (1)	Monotherapy	NSCLC
	Multi-kinase inhibitors				
	Navitoclax	Bcl-2, Bcl-x, Bcl-w	NCT02520778 (1)	Combination	NSCLC
	CT-707	ALK, FAK, Pyk2	NCT02695550 (1)	Monotherapy	NSCLC
	Famitinib	c-Kit, VEGFR2, PDGFR, VEGFR3, FLT1, FLT3	NCT02356991 (2)	Monotherapy	NSCLC
			NCT02364362 (1)	Combination	NSCLC
	MGCD516	VEGFR, PDGFR, DDR2, TRK and Eph families	NCT02219711 (1)	Monotherapy	NSCLC/SCLC
	Pexidartinib	Kit, FLT3, CAF1r	NCT02452424 (1/2)	Combination	NSCLC
	Anlotinib	VEGF1/2/3, FGFR2	NCT02388919 (2/3)	Monotherapy	NSCLC
	Entrectinib	NTRK1/2/3, ROS1, ALK	NCT02568267 (2)	Monotherapy	NSCLC
	ASP2215	Axl, FLT3	NCT02495233 (1/2)	Combination	NSCLC/SCLC
	PI3K/mTOR pathway inhibitors				
	MLN1117	PI3K	NCT02393209 (1/2)	Combination	NSCLC
	AZD8186		NCT01884285 (1)	Monotherapy	NSCLC
	LY3023414	PI3K, mTOR	NCT02443337 (2)	Combination	NSCLC
	Other miscellaneous target inhibitors				
	LEE011	CDK 4/6	NCT02292550 (1/2)	Combination	NSCLC
	Abemaciclib		NCT02308020 (2)	Monotherapy	NSCLC
			NCT02779751 (2)	Monotherapy	NSCLC
			NCT02079636 (1)	Combination	NSCLC
	INK128	TORC1/2	NCT02503722 (1)	Combination	NSCLC
	Alisertib	Aurora kinase inhibitor	NCT01471964 (1/2)	Combination	NSCLC
	Ibrutinib	BTK	NCT02321540 (1/2)	Monotherapy	NSCLC
	TAK-659	Syk	NCT02834247 (1)	Combination	NSCLC
	Pyrotinib	HER2	NCT02535507 (2)	Monotherapy	NSCLC
	EphB4-HSA	sEphB4	NCT02495896 (1)	Combination	NSCLC
	Ficlatuzumab	Hepatocyte growth factor (HGF)	NCT02318368 (2)	Combination	NSCLC
	AMG 479	IGFR-1	NCT01061788 (1)	Combination	NSCLC/SCLC
	MM-121	HER3	NCT02387216 (2)	Combination	NSCLC
	Defactinib	Focal adhesion kinase (FAK)	NCT02758587 (1/2)	Combination	NSCLC
	JNJ-42756493	FGFR	NCT02699606 (2)	Monotherapy	NSCLC
	INCB054828		NCT02393248 (1/2)	Monotherapy	NSCLC/SCLC
	LOXO-101	NTRK1/2/3	NCT02576431 (2)	Monotherapy	NSCLC/SCLC
	RXDX-101		NCT02097810 (1)	Monotherapy	NSCLC/SCLC
	Rh-endostatin		NCT02375022 (2)	Combination	NSCLC
	Cediranib		NCT02498613 (2)	Combination	NSCLC/SCLC
	GSK3052230	FGF ligand trap	NCT01868022 (1)	Combination	NSCLC/SCLC
	TRC105	Endoglin (CD105)	NCT02429843 (1)	Combination	NSCLC
	MEK162	MEK	NCT01859026 (1)	Combination	NSCLC
	PD-0325901		NCT02022982 (1/2)	Combination	NSCLC
	Selumetinib	RAS/RAF/MEK/ERK	NCT01586624 (1)	Combination	NSCLC
	Pacritnib	JAK2	NCT02342353 (1/2)	Monotherapy	NSCLC
	AT13387	Heat shock protein 90	NCT02535338 (1/2)	Combination	NSCLC
	AUY922		NCT01922583 (2)	Monotherapy	NSCLC
			NCT01854034 (2)	Monotherapy	NSCLC
	Galunisertib	TGFβ signaling	NCT02423343 (1/2)	Combination	NSCLC/SCLC
	MSC2156119J	MET	NCT01982955 (1/2)	Combination	NSCLC
	Ralimetinib	MAPK	NCT02860780 (1)	Combination	NSCLC/SCLC
	LTT462		NCT02711345 (1)	Monotherapy	NSCLC
	PF-06671008	P-cadherin	NCT02659631 (1)	Monotherapy	NSCLC/SCLC
	BGB324	Axl	NCT02424617 (1/2)	Combination	NSCLC
DNA repair	VX790	ATR	NCT02487095 (1/2)	Combination	SCLC
	Veliparib	PARP	NCT01386385 (1/2)	Combination	NSCLC
	Olaparib		NCT02498613 (2)	Combination	NSCLC/SCLC
Chemotherapy	Plinabulin	Tubulin-depolymerization	NCT02846792 (1/2)	Combination	NSCLC
			NCT02812667 (1)	Combination	NSCLC
	PT-112	Platinum based	NCT02884479 (1/2)	Combination	NSCLC/SCLC
	NC-6004	Micellar nanoparticle-encapsulated cisplatin	NCT02240238 (1/2)	Combination	NSCLC
	EC1456	Folic acid-tubulysin conjugate	NCT01999738 (1)	Monotherapy	NSCLC/SCLC
Oncolytic virus	CVA21	Coxsackievirus A21	NCT02043665 (1)	Monotherapy	NSCLC
			NCT02824965 (1)	Combination	NSCLC

*ALK* anaplastic lymphoma kinase, *ATR* ataxia telangiectasia and Rad3-related protein, *AXL* AXL receptor tyrosine kinase, *BTK* Bruton’s tyrosine kinase, *CDK 4/6* cyclin-dependent kinase 4/6, *CEACAM1* carcinoembryonic antigen-related cell adhesion molecule 1, *dUTPase* deoxyuridine triphophatase, *FAK* focal adhesion kinase, *FGF* fibroblast growth factor, *FLT1/3* fms-like tyrosine kinase 1/3, *c-Kit* proto-oncogene c-Kit, *Her2* human epidermal growth factor receptor 2, *HLA* human leukocyte antigen, *IGFR* insulin-like growth factor receptor, *JAK2* Janus kinase 2, *MAGEA3* melanoma-associated antigen 3, *MAPK* mitogen-activated protein kinase, *MEK* mitogen-activated protein kinase kinase, *mTOR* mammalian target of rapamycin, *NTRK3* neurotrophic tyrosine kinase, *PARP* poly ADP ribose polymerase, *PDGFR* platelet-derived growth factor receptor, *PI3K* phosphatidylinositide 3-kinases, *sEphB4* soluble extracellular domain of EphB4, *Syk* spleen tyrosine kinase, *VEGFR* vascular endothelial growth factor receptor, *VISTA* V-domain Ig suppressor of T cell activation, *WT1* Wilms’ tumor protein

## Conclusions

Oncology is changing at a fast pace, and improved outcomes are being observed in most human malignancies. Until now, lung cancer has lagged behind, but novel targeted agents and immunotherapies have shown promising results for this common and aggressive cancer. The rapid development of novel agents targeting those molecular driver alterations in lung cancer will likely further improve the clinical outcomes. The combination of agents that target non-overlap pathways will likely provide additive or synergistic activities. Many studies are ongoing to test new targets for immunotherapy such as inhibitory molecules TIM-3, LAG-3, IDO (Indoleamine-pyrrole 2,3-dioxygenase), BTLA (B- and T-lymphocyte attenuator), adenosine, VISTA (V-domain immunoglobulin containing suppressor of T cell activation), and stimulatory molecules such as 4-1BB, OX40, CD40, and CD27. A great number of clinical studies are underway to test the clinical activities of various immunotherapy combination strategies in different settings. However, there are challenges to conquer. Treatment with target agents inevitably leads to drug resistance. Understanding the resistance mechanisms and developing novel agents or strategies targeting the resistant tumors are largely need. Although immunotherapy has shown durable response in some patients, the majority of patients do not benefit. Immunohistochemistry staining of PD-L1 on tumor cells is extensively studied but still remains as unperfect biomarker. Discovery of new biomarkers or combination of biomarkers are required to guide the selection of patients who most likely benefit from treatment to avoid unnecessary costs and toxicities. Many pre-clinical and clinical studies are ongoing to address these needs.

## Abbreviations

ALK: Anaplastic lymphoma kinase
DCR: Disease control rate
EGFR: Epidermal growth factor receptor
NSCLC: Non-small cell lung cancer
ORR: Overall response rate
PD: Progressive disease
PR: Partial response
SD: Stable disease
